# Copy number variation in the susceptibility to systemic lupus erythematosus

**DOI:** 10.1371/journal.pone.0206683

**Published:** 2018-11-28

**Authors:** Fernanda Bueno Barbosa, Milena Simioni, Cláudia Emília Vieira Wiezel, Fábio Rossi Torres, Miriam Coelho Molck, Melvin M. Bonilla, Tânia Kawasaki de Araujo, Eduardo Antônio Donadi, Vera Lúcia Gil-da-Silva-Lopes, Bernardo Lemos, Aguinaldo Luiz Simões

**Affiliations:** 1 Department of Genetics, Ribeirão Preto Medical School, USP, Ribeirão Preto, SP, Brazil; 2 Department of Medical Genetics, Faculty of Medical Sciences, UNICAMP, Campinas, SP, Brazil; 3 Department of Environmental Health, Harvard T.H. Chan School of Public Health, Boston, MA, United States of America; 4 Division of Clinical Immunology, Department of Medicine, Ribeirão Preto Medical School, USP, Ribeirão Preto, SP, Brazil; Peking University First Hospital, CHINA

## Abstract

Systemic lupus erythematosus (SLE) is an autoimmune disease with a strong genetic component and etiology characterized by chronic inflammation and autoantibody production. The purpose of this study was to ascertain copy number variation (CNV) in SLE using a case-control design in an admixed Brazilian population. The whole-genome detection of CNV was performed using Cytoscan HD array in SLE patients and healthy controls. The best CNV candidates were then evaluated by quantitative real-time PCR in a larger cohort or validated using droplet digital PCR. Logistic regression models adjusted for sex and ancestry covariates was applied to evaluate the association between CNV with SLE susceptibility. The data showed a synergistic effect between the *FCGR3B* and *ADAM3A* loci with the presence of deletions in both loci significantly increasing the risk to SLE (5.9-fold) compared to the deletion in the single *FCGR3B* locus (3.6-fold). In addition, duplications in these genes were indeed more frequent in healthy subjects, suggesting that high *FCGR3B*/*ADAM3A* gene copy numbers are protective factors against to disease development. Overall, 21 rare CNVs were identified in SLE patients using a four-step pipeline created for identification of rare variants. Furthermore, heterozygous deletions overlapping the *CFHR4*, *CFHR5* and *HLA-DPB2* genes were described for the first time in SLE patients. Here we present the first genome-wide CNV study of SLE patients in a tri-hybrid population. The results show that novel susceptibility loci to SLE can be found once the distribution of structural variants is analyzed throughout the whole genome.

## Introduction

Systemic lupus erythematosus (SLE, MIM 152700) is an autoimmune polygenic disease characterized by local or systemic inflammation from the production of autoantibodies and immune complex deposition in several tissues [1]. SLE has a wide range of clinical manifestations such as malar rash, discoid lesions, nephritis, and arthritis [2]. SLE is more prevalent in women than in men at a rate of 9:1, and onset predominantly during childbearing age [3]. In general, it is less prevalent in European ancestry populations than in African-Americans, African-Caribbean, Asians, and Hispanics [4].

In addition to the genetic component; hormonal, environmental, epigenetic and immunological factors contribute to the complex etiology of SLE [5]. High heritability and increased concordance rates were identified in monozygotic twins (24–57%) compared with dizygotic twins or full siblings (2–5%), suggesting that SLE has a complex genetic basis with sizable genetic and environmental components [6, 7]. Linkage and genome-wide association studies conducted in cohorts of patients with SLE have confirmed that *HLA* and other loci are associated with SLE [8]. This method has also been useful in identifying new candidate single nucleotide polymorphisms (SNPs) correlated with the disease [9, 10].

In addition to SNPs, genomic segments that vary in copy number in relation to a reference genome (denoted as copy number variations, or CNVs, and typically greater than 50 bp [11]) have been associated with susceptibility to autoimmune diseases, including SLE [2]. Well-documented CNVs that increase risk for SLE include deletions in *C4* [12] and *FCGR3B* [13] genes, while CNVs in other genes were associated with SLE in single-population studied, e.g., *TLR7* [14], *DEFB4* [15], *RABGAP1L* [16] and *HLA-DRB5* [17].

The small number of large-scale studies relating CNVs to SLE remains a significant gap in the genetic analysis of the disease [16, 18]. Additionally, the ancestral composition of populations can often modify the results of association tests, such that the study of admixed populations can produce different or even conflicting results compared with those reported in the literature [19, 20].

Based on the hypothesis that new SLE-related loci remain to be discovered using CNV approach, this study has evaluated the role of structural variation in SLE through genome-wide screening in Brazilian SLE patients.

## Materials and methods

### DNA samples

The total case group comprised 135 unrelated SLE patients treated at the Collagen Disease Outpatient Clinic of the University Hospital at the Ribeirão Preto Medical School (HCFMRP, USP) in Brazil. All patients fulfilled the American College of Rheumatology revised criteria for SLE diagnosis [21]. The healthy control (HC) cohort includes 200 healthy unrelated subjects resident in São Paulo state, Brazil. DNA was extracted from the blood samples of SLE patients and control subjects using salting-out method [22] and QIAamp DNA Blood Maxi Kit (QIAGEN, Hilden, Germany), respectively.

For the array analysis, we selected a subgroup of 23 lupus nephritis patients (18 female, mean age at diagnosis: 31 ± 13 years) from the total of 135 SLE patients (125 female, mean age at diagnosis: 32 ± 12 years). The frequency of clinical characteristics for the group and subgroup of SLE patients was, respectively: (1) nephritis: 61%/100%, (2) arthritis: 61%/74%, (3) malar rash: 38%/48%, (4) oral ulcers: 13%/13%, (5) photosensitivity: 40%/52%, (6) convulsions and/or psychoses: 21%/30%, (7) hematologic disorder (hemolytic anemia or leukopenia < 4.000/mm^3^, lymphopenia < 1.500/mm3, and/or thrombocytopenia < 150.000/mm^3^): 57%/52%, (8) immunologic disorder (anti-dsDNA, anti-Sm, and/or anti-phospholipid): 73%/78%.

### Ethics

The study design was approved by the Research Ethics Committee of FMRP/USP (CAAE: 03199712.0.0000.5440) and FCM/UNICAMP (CAAE: 03199712.0.3001.5404). All subjects enrolled in this research have signed the consent form approved by the ethics committees.

### Cytoscan HD array

The genome-wide human Cytoscan HD array (Affymetrix, CA, USA) was used to detect CNVs in SLE patients (n = 23) and healthy controls (n = 110) according to the manufacturer's protocol. Scanned data files were generated using Affymetrix GeneChip Command Console software v. 1.2 and analyzed by Affymetrix Chromosome Analysis Suite (ChAS) software v. 3.0.

### CNV detection

To calculate copy number regions throughout the genome the data were normalized to baseline intensities according to an internal reference model of ChAS software that comprised 270 HapMap samples and 96 other healthy subjects from BioServe Biotechnologies (BioServe, Beltsville, USA). CNVs regions were mapped according to the human reference sequence version GRCh37/hg19. The number of consecutive probes required defining each deletion or duplication and was limited to a minimum of 25/ 50 consecutive probes, respectively. After variant detection by ChAS, CNV distribution per subjects and chromosomes was analyzed using Plink v.1.07 [23].

#### Determination of CNV regions

Plink was used to evaluate the recurrence of common CNVs, CNV regions (CNVRs), which are the union of overlapping CNVs among subjects [24]. Duplications and deletions were analyzed separately and classified as gain- or loss-type CNVRs.

### Rare CNVs

A four-step pipeline was created for the identification of rare variants (population frequency < 1%) based on the Brazilian/HapMap population frequencies, public databases of genomic variants and CNV detection by two different algorithms: ChAS and Nexus Copy Number 8.0 (BioDiscovery, CA, USA) (S1 Fig).

### CNVs located in genes with functional relevance to SLE

Using the *cytoregion* tool from ChAS software, three gene lists were created: (1) genes overlapping deletion or duplication-type CNVs described in association with SLE, (2) genes previously associated to SLE in linkage analysis and/or in genome-wide association studies, (3) genes related to autoimmunity. For this analysis, the number of consecutive markers for detecting deletions and duplications that overlap the genes included in the three lists was reduced from 25/50 to 15/15.

## Validation of CNVs using target-specific methodology

For all copy number genes selected for validation by target-specific methodology, we designed primers using Primer3Plus [25], verified their specificity with *in silico PCR* tool available from UCSC Genome Browser [26], and purchased them from Eurofins Genomics (Louisville, KY, USA) or Sigma-Aldrich (St Louis, MO, USA). Primer sequences are listed in S1 Table. *FOXP2* or *PAX6* were used as reference genes for diploid copy number.

The CNVR encompassing the *ADAM3A* gene was selected for validation using quantitative real-time PCR (qPCR) in a larger case-control cohort. In addition to *ADAM3A* target, an individual CNV assay was conducted for *FCGR3B* gene since its coverage is very low in the Cytoscan HD chip (S3 Table). The *ADAM3A* and *FCGR3B* gene copy number genotyping was performed by SYBR Green-based genomic qPCR in cases (n = 135) and controls (n = 200) using the StepOne Plus Real-Time PCR System according to the manufacturer's protocol (Applied Biosystems, CA, USA). All experiments were designed using technical triplicates for each sample. The reference and target genes for each sample were ran in the same 96-well plate to avoid introducing experimental bias. The copy number of the target gene in each test sample was determined by the ΔΔCT-based relative quantification method [27].

Droplet digital PCR (ddPCR) was used as a target-specific methodology to validate three heterozygous deletions (CN = 1) overlapping the *CFHR4*, *CFHR5*, *HLA-DPB2* genes, and a heterozygous duplication (CN = 3) encompassing the *LDHB*, *KCNJ8*, *ABCC9*, *CMAS* and *ST8SIA1I* genes (S4 Table). The ddPCR experiments were performed according to the manufacturer's protocol (Bio-Rad Laboratories, CA, USA). As the initial step, we treated all genomic DNA samples with *HindIII* restriction enzyme for 2 h at 37°C and then proceed to EvaGreen ddPCR assay. We calculated copy number using the QuantaSoft Pro software (Bio-Rad Laboratories, CA, USA). The error reported for a single well was the Poisson 95% confidence interval (95% CI). We used the automated clustering analysis for both target and reference and then calculated the final copy number as two times the ratio of target concentration versus reference concentration. A reference sample, expected to be in a diploid status in both target and reference genes was used as an internal control of the reactions.

### Ancestry inference

Due to tri-hybrid composition of the Brazilian population, a panel of 345 ancestry informative markers based on SNP data from the array was used to infer the proportion of European, African and Amerindian ancestries of each SLE and control subjects [28]. These estimates were used as covariates in logistic regression models for the association between CNV with SLE susceptibility.

### Statistical analysis

Statistical analyses were performed using the computing environment R version 3.1.1 (http://www.r-project.org/). The call and size of CNV was compared between SLE patients and controls using logistic regression models adjusted for sex and ancestry covariates, while Student's t-test was performed to compare the call and size of CNV per chromosome between case and control groups.

## Results

### Characteristics of CNVs

**Fig 1:**
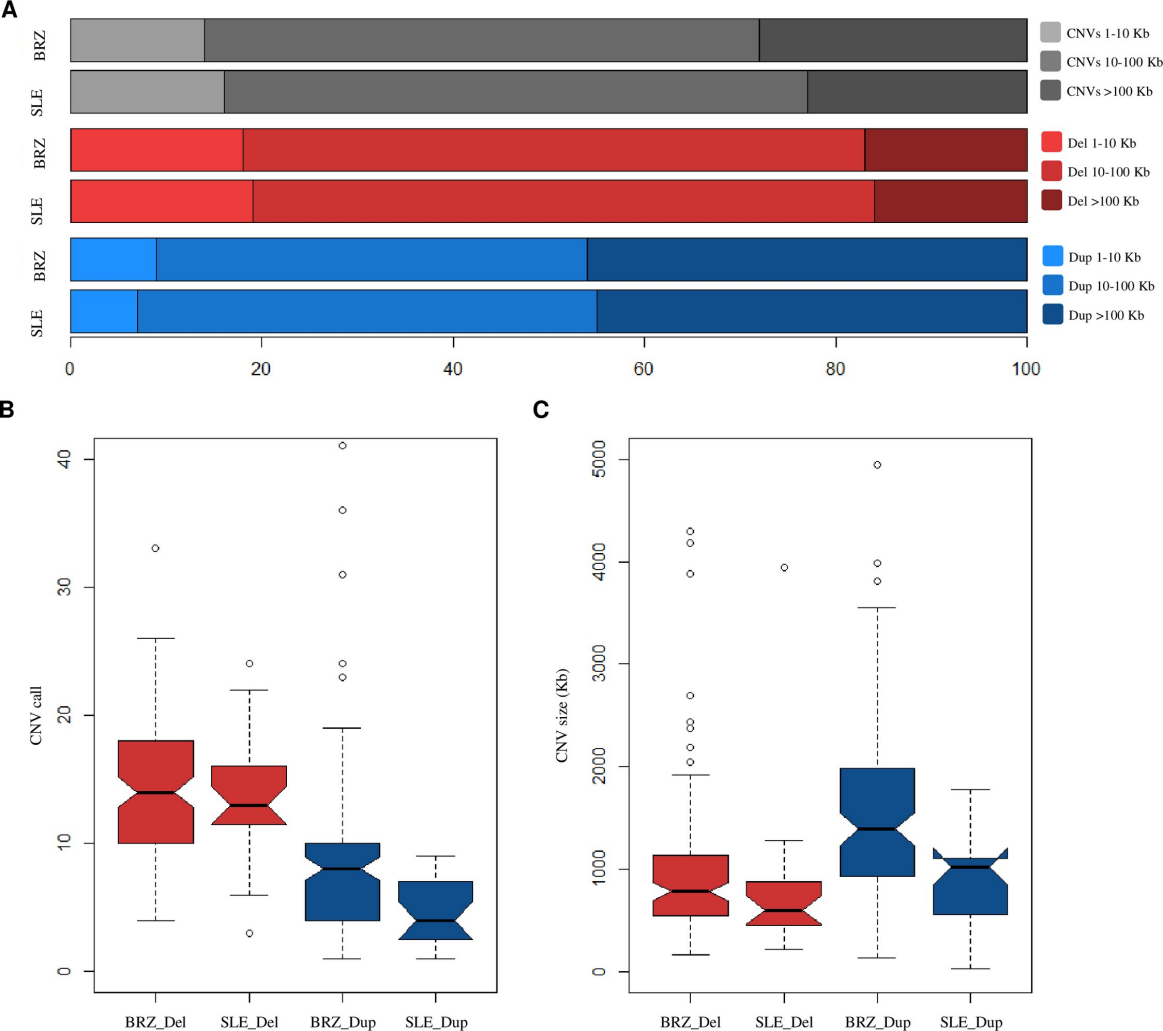
Deletion and duplication profiles in the systemic lupus erythematosus (SLE) patients and Brazilian controls. Distribution of copy number variation (CNVs), deletions (del) and duplications (dup) according to the size ranges in SLE patients and Brazilian healthy individuals (BRZ) (A). Notched box plot comparing the distribution of call (B) and size (C) of deletions and duplications in SLE patients and controls. The boxes represent the interquartile range with black lines indicating the median of the data and the leaked circles symbolizing outliers. The notches exhibit a 95% confidence interval around the median. According to Chambers (1983), if there is no overlap between the notches, there is evidence that the differences between the groups evaluated are significant, as we observed in the call and size of duplications between cases and controls.

Genomic screening using the Cytoscan HD array found 447 CNVs in 23 patients and 2652 CNVs in 110 controls. An average of 19 CNVs were identified per patient (SD *=* 10, range 5–55 CNVs), while in controls the average was 24 CNVs (SD *=* 11, range 7–74 CNVs) (S2 Fig). Deletions were 1.6–3.2 times more frequent than duplications, corresponding to 62% of CNVs identified in controls and 76% in SLE patients; however the average deletion size was 3.2–4.0 smaller than the average duplication size both control and case groups, respectively (Fig 1A).

Case-control comparisons with logistic regression models adjusted for sex and ancestry showed that duplications were present in smaller number (*p* = 0.001, OR = 0.7 [95% CI, 0.6–0.9]) in SLE patients than in healthy subjects. No evidence for differences in the total number (*p* = 0.552, OR = 1.0 [95% CI, 0.9–1.0]) and size (*p* = 0.341, OR = 1.0 [95% CI, 1.0–1.0]) of deletions were observed between case and control groups (Fig 1B).

**Fig 2:**
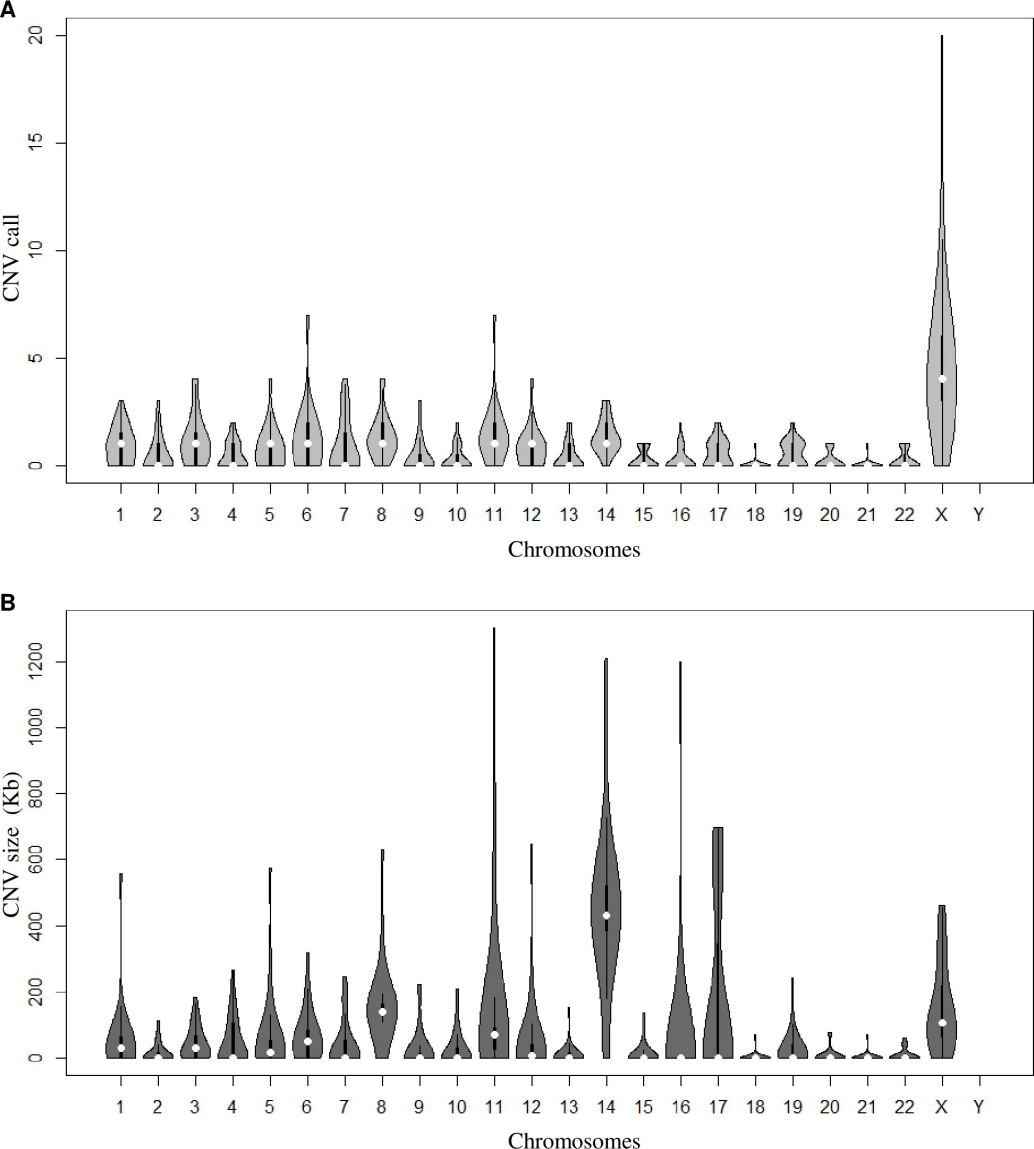
Chromosome distribution of copy number variation (CNV) in systemic lupus erythematosus (SLE) patients. Violin plot (R package: vioplot v. 0.2) illustrating the distribution of the data and its probability density for call (A) and size (B) of the total number of CNVs per chromosome. The thicker black bars in the center represent the interquartile range, while the thin black line extended from it represents the 95% confidence intervals around the median (white dot).

CNVs were detected in all autosomes and in the X chromosome. In both groups, the X chromosome showed the highest number of CNVs, representing an average of 10 X-linked CNVs per SLE patient (SD *=* 4, range 0–20 CNVs) and 6 CNVs per control (SD *=* 5, range 0–29 CNVs). On the other hand, the larger CNVs were concentrated on chromosome 14, and showed more CNVs (*p* = 0.020) of larger size (*p* = 0.004) in SLE patients compared to controls. Chromosome 1 also showed a higher number of CNV calls per patient compared to controls (*p* = 0.041) (Fig 2).

### Synergic effect of deletion in the *FCGR3B* and *ADAM3A* genes

In the CNVRs analysis performed from the Cytoscan HD array data (23 SLE and 110 controls), a deletion (CN < 2) partially encompassing the *ADAM5* gene and entirely overlapping the *ADAM3A* gene was identified as a potential candidate for increased susceptibility to SLE (*p* = 0.0352; OR = 3.3 [95% CI, 1.0–14.1]) (S3 Fig and S3 Table). Based on this result, we designed a qPCR assay using the *ADAM3A* as a target gene in order to validate the association in a larger population of SLE patients (n = 135) and controls (n = 200). The association of the deletion in the *ADAM3A* gene with SLE was not replicated using qPCR in the larger sample population (*p* = 0.99, OR = 1.0 [95% CI, 0.6–1.8]). However, in agreement with our expectations we observed that *ADAM3A* duplication was statistically lower in SLE patients than in controls (*p* = 1.23 x 10^−2^, OR = 0.2 [95% IC, 0.1–0.7]) (S4 Fig), suggesting that gains in the *ADAM3A* gene are a protective factor for the development of SLE.

**Fig 3:**
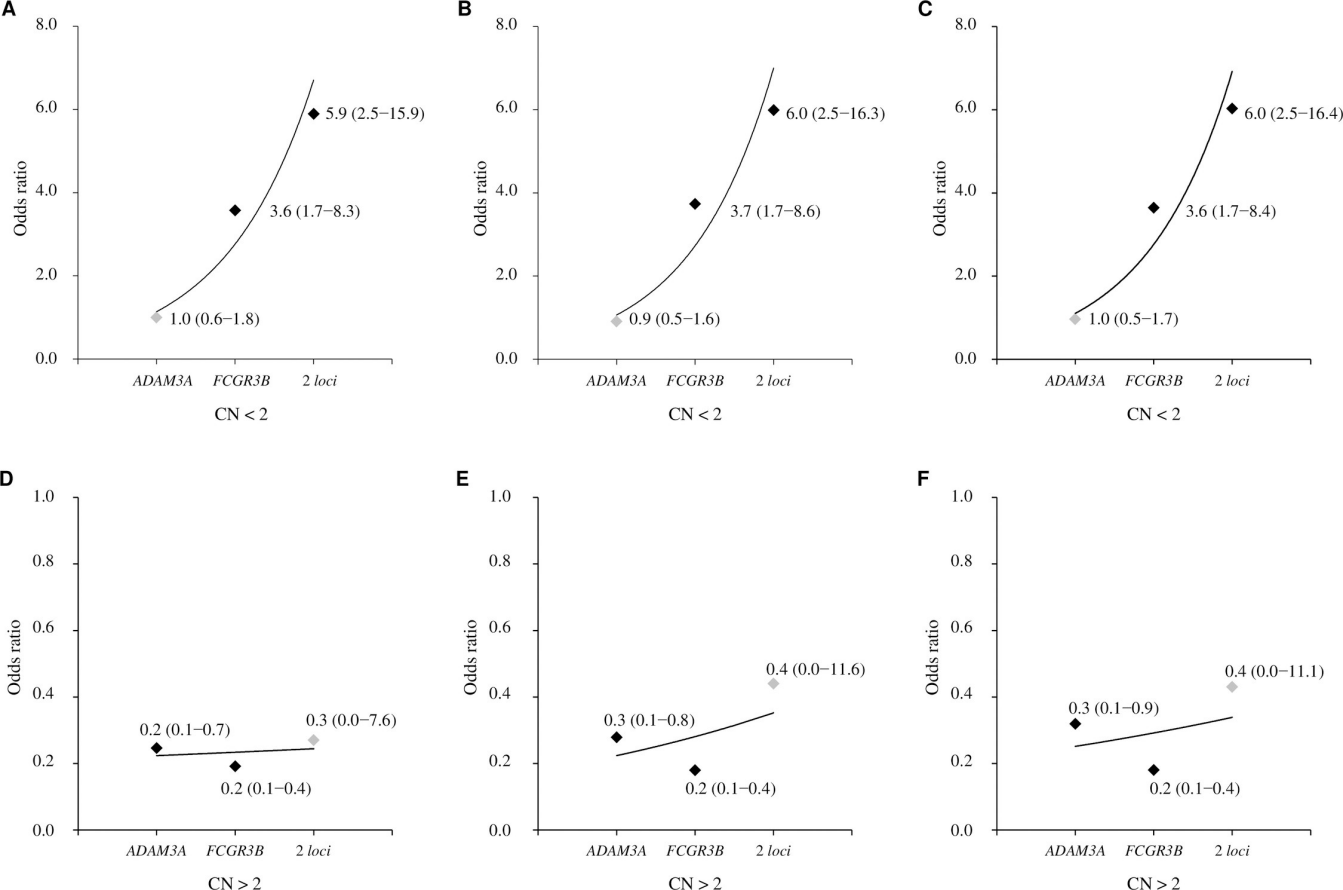
Copy number variation in *ADAM3A* and *FCGR3B* genes in systemic lupus erythematosus (SLE). The risk of SLE in relation to the copy number (CN) < 2 (deletions at *ADAM3A*, *FCGR3B*, or 2 *loci*) and CN > 2 (duplications at *ADAM3A*, *FCGR3B*, or 2 *loci*), was estimated relative to subjects without any variation in both loci (diploid status at 2 *loci*) as a reference. The point estimates values represent odds ratios with 95% confidence interval according to adjusted logistic regression models for sex and African (A, D), European (B, E) and Amerindian (C, F) ancestries.

In an independent CNV assay, we showed that deletion in the *FCGR3B* gene is associated with increased susceptibility to SLE (*p* = 1.66 x 10^−3^, OR = 3.6 [95% CI, 1.7–8.3]), while duplication in this gene is a protective factor for the development of SLE (*p* = 1.55 x 10^−4^, OR = 0.2 [95% CI, 0.1–0.4]) (S4 Fig). Evaluating the simultaneous presence of the deletion in both *FCGR3B* and *ADAM3A* loci, we observed that their joint presence contributes to the increase of susceptibility to SLE (*p* = 1.4 x 10^−4^, OR = 5.9 [95% CI, 2.5–15.9]) when compared to a deletion only in the *FCGR3B* gene. This suggests a genetic interaction between these loci in SLE. The values presented above correspond to the logistic regression models adjusted for sex and African component. The adjusted models for the European and Amerindian components show similar results to the African one (Fig 3).

### Rare CNVs and CNVs overlapping genes with functional relevance to SLE

**Fig 4:**
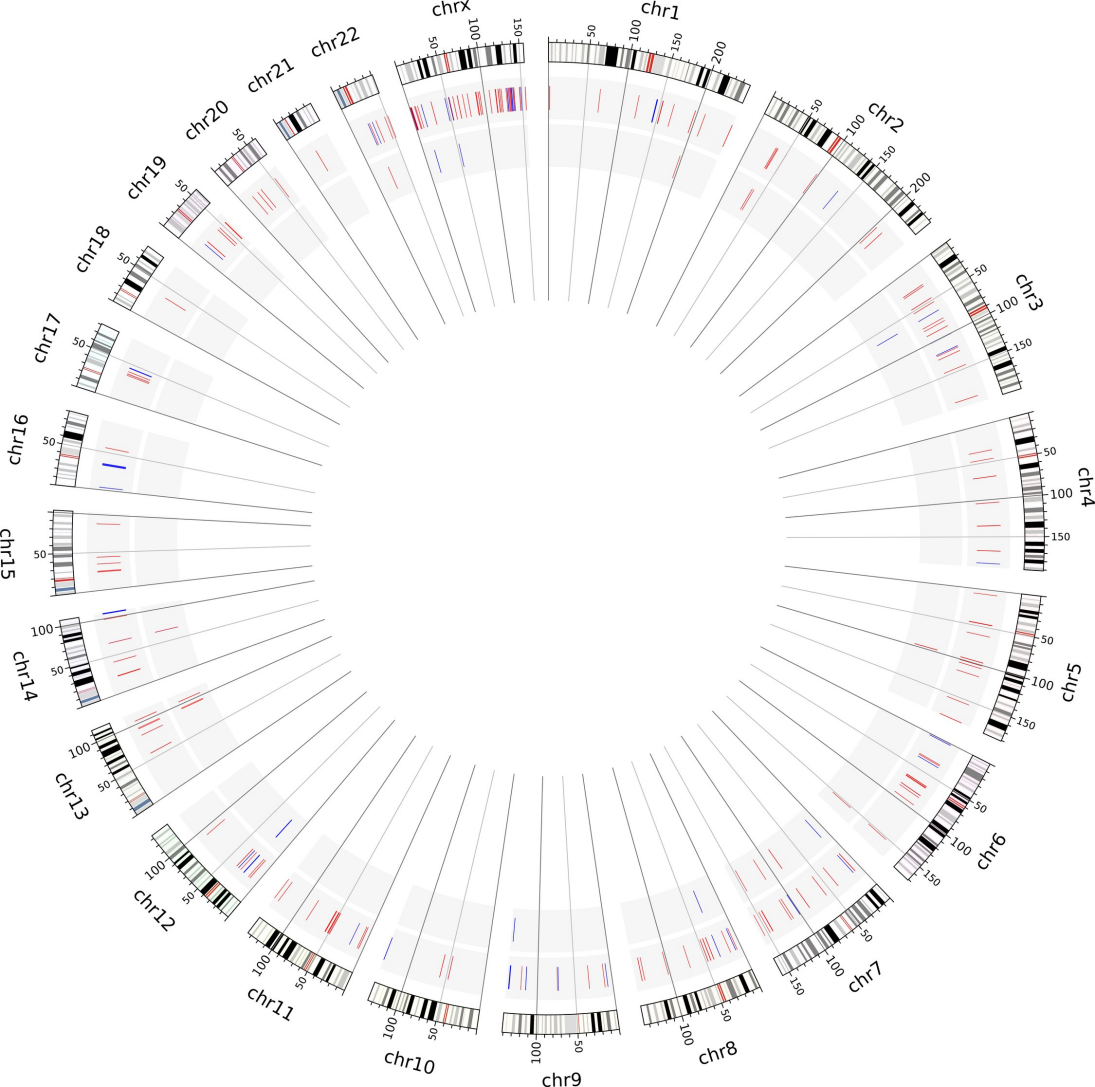
Circo plot showing the distribution of copy number variation (CNV) in the genome of systemic lupus erythematosus (SLE) patients. The largest circos represent the 22 autosomal chromosomes and the X chromosome according to the GRCh37/hg19 version of the human genome, followed by the set of CNVs identified in SLE patients and the rare CNVs. Deletions and duplications are represented in red bars and blue bars.

We applied the four-step pipeline to identify rare CNVs in the set of 447 CNVs detected in the 23 SLE patients. Comparing all CNVs identified in SLE patients with the 2652 CNVs reported for the control group, we identified 88/447 variants not present in the control subjects, i.e. exclusive CNVs of SLE. After filtering using population data of healthy subjects from the HapMap project and then the Database of Genomic Variants, we identified 67/88 CNVs and 49/67 CNVs as exclusive to our SLE sample. As a final step, we used an alternative algorithm (Nexus Copy Number) to detect CNVs in SLE patients. Considering only variants identified using both ChAS and Nexus Copy Number software, our four-step pipeline resulted in the detection of 21 rare CNVs that are exclusive to our SLE sample (Fig 4 and Table 1).

**Table 1:** Description of rare copy number variation identified in systemic lupus erythematosus (SLE) patients from the four-step pipeline application.

Genomic location (GRCh37/hg19)	Genes	Type	Size (Kb)	SLE ID	Pop freq
chr1:196964969–196987806	*CFHR5*	Del	23	SLE005	–
chr2:34219274–34313413	–	Del	94	SLE009	< 1%
chr2:36932842–36964864	*VIT*	Del	32	SLE010	–
chr3:65517399–65595008	*MAGI1*	Dup	78	SLE015	< 1%
chr5:96625630–96865984	–	Del	240	SLE014	–
chr6:152303218–152311895	*ESR1*	Del	9	SLE003	< 1%
chr7:11983316–12078163	–	Dup	95	SLE005	< 1%
chr7:85522989–85709099	–	Del	186	SLE003	< 1%
chr7:114209440–114215739	*FOXP2*	Del	6	SLE023	–
chr7:146859536–146868357	*CNTNAP2*	Del	9	SLE003	–
chr8:30079990–30202711	*MIR548O2*	Dup	123	SLE018	–
chr9:137354612–137529641	–	Dup	175	SLE023	< 1%
chr12:21796951–22445614	*LDHB*, *KCNJ8*, *ABCC9*, *CMAS*, *ST8SIA1*	Dup	649	SLE018	< 1%
chr13:93378273–93386333	*GPC5*	Del	8	SLE023	–
chr13:94506273–94515199	*GPC6*, *GPC6-AS2*	Del	9	SLE023	< 1%
chr13:104521177–104647267	–	Del	126	SLE013	–
chr14:67111441–67148873	*GPHN*	Dup	37	SLE011	–
chr14:67193022–67217571	*GPHN*	Del	25	SLE016	< 1%
chr22:25066486–25112325	–	Del	46	SLE006	< 1%
chrX:15723836–15831401	*CA5B*, *INE2*, *ZRSR2*	Dup	108	SLE018	–
chrX:52833688–52904643	*XAGE5*, *XAGE3*	Dup	71	SLE017	–

chr = chromosome; Del = deletion; Dup = duplication; ID = identification; Pop freq = population frequency.

Gene list analysis was used to evaluate the presence of genes with functional relevance to SLE within the interval of CNV, and revealed CNVs overlapping 8/153 genes in SLE group (S2 Table).

Based on population frequency of CNV, deletions in *CFHR4*, *CFHR5*, *STAT4* and *HLA-DPB2* genes identified using Cytoscan HD array in the rare and functional relevant CNVs were selected for validation by ddPCR. In addition to these selected CNVs, a rare duplication of 649 Kb in size encompassing five genes (*LDHB*, *KCNJ8*, *ABCC9*, *CMAS* and *ST8SIA1I*) was also selected for target-specific validation (S3 Fig). The genotypes were confirmed for 4/5 CNVs selected for ddPCR validation. Therefore, we described heterozygous deletions (CN = 1) in *CFHR4*, *CFHR5*, *HLA-DPB2* genes, and a heterozygous duplication (CN = 3) encompassing the *LDHB*, *KCNJ8*, *ABCC9*, *CMAS* and *ST8SIA1I* genes (S5 Fig). Heterozygous deletion in the *STAT4* gene was not confirmed by ddPCR.

## Discussion

Despite remarkable progress in the identification of loci-specific CNVs associated with SLE, questions regarding the role of structural variation in genetic variability and SLE susceptibility have remained [13, 15, 16, 29]. Here we present the first genome-wide CNV study of SLE patients in a tri-hybrid population. As crucial step to obtain reliable association results for admixed cohorts [20, 28], we performed adjustment for population stratification for all CNV statistical comparisons between case and control groups. We reported the synergistic effect of common multi-allelic CNVs increasing the risk for SLE compared to the variation in a single locus, further supporting the involvement of multi-loci deletions in the etiology of the disease. Our study incorporate two CNV calling algorithms and added data of other healthy subjects in external populations and databases with the aim of increasing stringency and improving the sensitivity and specificity of detecting rare CNVs throughout the genome.

The higher total number of deletions in whole-genome in relation to duplications and the smaller average size of losses compared to gains found in this study for both case and control groups, were also reported for Korean women SLE patients and their respective controls [16], as well as for healthy subjects from the Database of Genomic Variants [11]. The excess of X-linked CNVs observed here also corroborates methodologically similar reports, suggesting that there is predominance of variants in the X chromosome over the other chromosomes for both disease and control groups [18, 30]. These findings that found no evidence of differences in CNV distribution in disease and non-disease cohorts underscores the need to search for loci-specific CNVs as causal variants in the susceptibility and severity of complex diseases for CNV-phenotype associations, instead of focusing in the total burden of structural variants.

The putative association of low copy number of *FCGR3B* gene with an increased risk for SLE previously identified in African, Chinese and European ancestry populations [31] was replicated in our Brazilian cohort. The data corroborates the association of this gene with the disease even in an admixed population such as the one presented here. As *FCGR3B* is involved in the recruitment of polymorphonuclear neutrophils to sites of inflammation and clearance of immune complexes [31], losses in this gene could result in the reduction of neutrophil trafficking to inflammatory lesions and in a decrease in the ability to control immune response [32]. In addition to the 3.6-fold increase in the susceptibility of developing SLE observed in the single *FCGR3B* deletion, an additive effect was observed when there was in a deletion of both *FCGR3B* and *ADAM3A* genes, leading to a 5.9-fold increase risk for SLE. Similar synergistic effect of losses in three other loci encompassing the *RABGAP1L*, *C4*, and a region on chromosome 10q21 with no genes in the interval, which resulted in a 5.5-fold increase in the risk for developing SLE compared to deletions in any loci [16]. The observations are in agreement with the hypothesis that the combined effect of multiple dosage-sensitive genomic regions may lead to the predisposition to the disease.

Previous reports associated SLE with SNPs [8] and CNVs mapped in the major histocompatibility complex region, i.e., duplications in *HLA-DRB5* [17] and deletions in complement 4 (C4) gene [29, 33]. Here we report for the first time identified in SLE patients three heterozygous deletions overlapping HLA (*HLA-DPB2*) and complement-related genes (*CFHR4* and *CFHR5*). The deletions were identified using microarray screening followed by target-specific validation. Although the ability to attribute pathogenicity to a particular CNV remains limited, deep analysis of candidate risk loci harboring losses in the *HLA-DRB2* and other HLA genes could provide one possible update for the unexplained genetic disease susceptibility. SNPs in complement factor H (CFH) genes, a key regulator of the alternative complement pathway, have also been associated with SLE [34]. Our findings suggest new insights into the pathogenic mechanisms of complement factor H in SLE involving changes in copy number in the factor H-related genes, highlighting the homeostatic balance between *CFH-CFHR* genes as critical to maintain regulation in complement activation [34]. Since *CFHR4* gene plays a key role in regulating complement activation and opsonization on biological surfaces by interacting with C-reactive protein [35], deletion in this gene could lead to reduced protein binding and thus would limit its ability to inhibit inflammation, facilitating SLE development. The involvement of *CFHR5* in renal diseases, e.g. *CFHR5* nephropathy [36], has particular interest in view of the SLE patient who present the rare deletion in *CFHR5* here identified has lupus nephritis, suggesting that losses in this gene may compromise renal functioning and indicating that this variation may be related to lupus nephritis development.

## Conclusions

Here we show that novel susceptibility loci for SLE can be identified using large-scale evaluation of CNVs. This was the first-time identification of three heterozygous deletions encompassing the *CFHR4*, *CFHR5* and *HLA-DPB2* genes related to this disease. Additionally, a set of other rare CNVs of SLE patients were reported. We also showed the synergistic effect of deletion in both *FCGR3B* and *ADAM3A* genes increasing the risk to SLE. The detection of rare and common CNVs in functionally relevant genes may elucidate how lupus is triggered and clarify the relationship between clinical manifestations and biological pathways that underlie disease progression. The evaluation of the fine-scale architecture of CNV regions, as well as the prediction of pathogenicity of long segments encompassing several variants found in homozygosity, would contribute to understanding how risk loci harboring CNVs segments acts on the etiology of SLE.

## Supporting information

S1 Fig Steps for the detection of rare copy number variation (CNVs) and CNVs overlapping genes with functional relevance for systemic lupus erythematosus (SLE) etiology. (TIF)

S2 Fig Distribution of copy number variation (CNV) in the human genome. Histogram showing the frequency of each class of CNV call in systemic lupus erythematosus (SLE) patients (A) and in Brazilian controls (B). Kernel density plot showing the non-parametric distribution of probability density curve of CNVs in SLE patients (C) and in Brazilian controls (D).
(TIF)

S3 Fig Venn diagram showing the genes that overlap copy number variation (CNV) segments belonging to the lists of CNV regions (CNVRs), rare CNVs and CNVs located in genes with functional relevance to systemic lupus erythematosus. Genes in bold highlight those selected for further validation of the copy number status by target-specific methodology.
(TIF)

S4 Fig Distribution of copy number variation (CNV) genotypes according quantitative real-time PCR. Frequency of copy number (CN), deletions (del) and duplications (dup) in *ADAM3A* (A, B) and *FCGR3B* (C, D) genes in systemic lupus erythematosus (SLE) and Brazilian control (BRZ) groups.
(TIF)

S5 Fig Copy number variation genotypes obtained by droplet digital PCR. Charts showing the copy number (CN) for each SLE patient, confirming heterozygous deletions of *CFHR4* in SLE002 patient (A), *CFHR5* in SLE005 patient (B), HLA-DPB2 in SLE019 patient (C), and heterozygous duplication involving the *LDHB*, *KCNJ8*, *ABCC9*, *CMAS* and *ST8SIA1* genes in the SLE018 patient (D). In all cases, the reference gene (*FOXP2*) shows invariable diploid status in the subjects analyzed. The reference sample confirmed the diploid copy number for both target and reference genes.
(TIF)

S1 Table Primer sequences used in quantitative real-time PCR (qPCR) and droplet digital PCR (ddPCR). (DOCX)

S2 Table Description of copy number variation identified in systemic lupus erythematosus (SLE) patients overlapping genes with functional relevance to SLE. (DOCX)

S3 Table Description of copy number variation regions (CNVRs) showing significant difference in frequencies between systemic lupus erythematosus (SLE) patients and Brazilian controls. (DOCX)

S4 Table Description of copy number variation (CNVs) and copy number variation regions (CNVRs) selected for validation by target-specific methodology. (DOCX)
