# Supplementary figures and images for: Copy number variation in the susceptibility to systemic lupus erythematosus
Source: PLoS One. 2018 Nov 28;13(11):e0206683. doi: 10.1371/journal.pone.0206683 (PMC6261406; doi:10.1371/journal.pone.0206683)

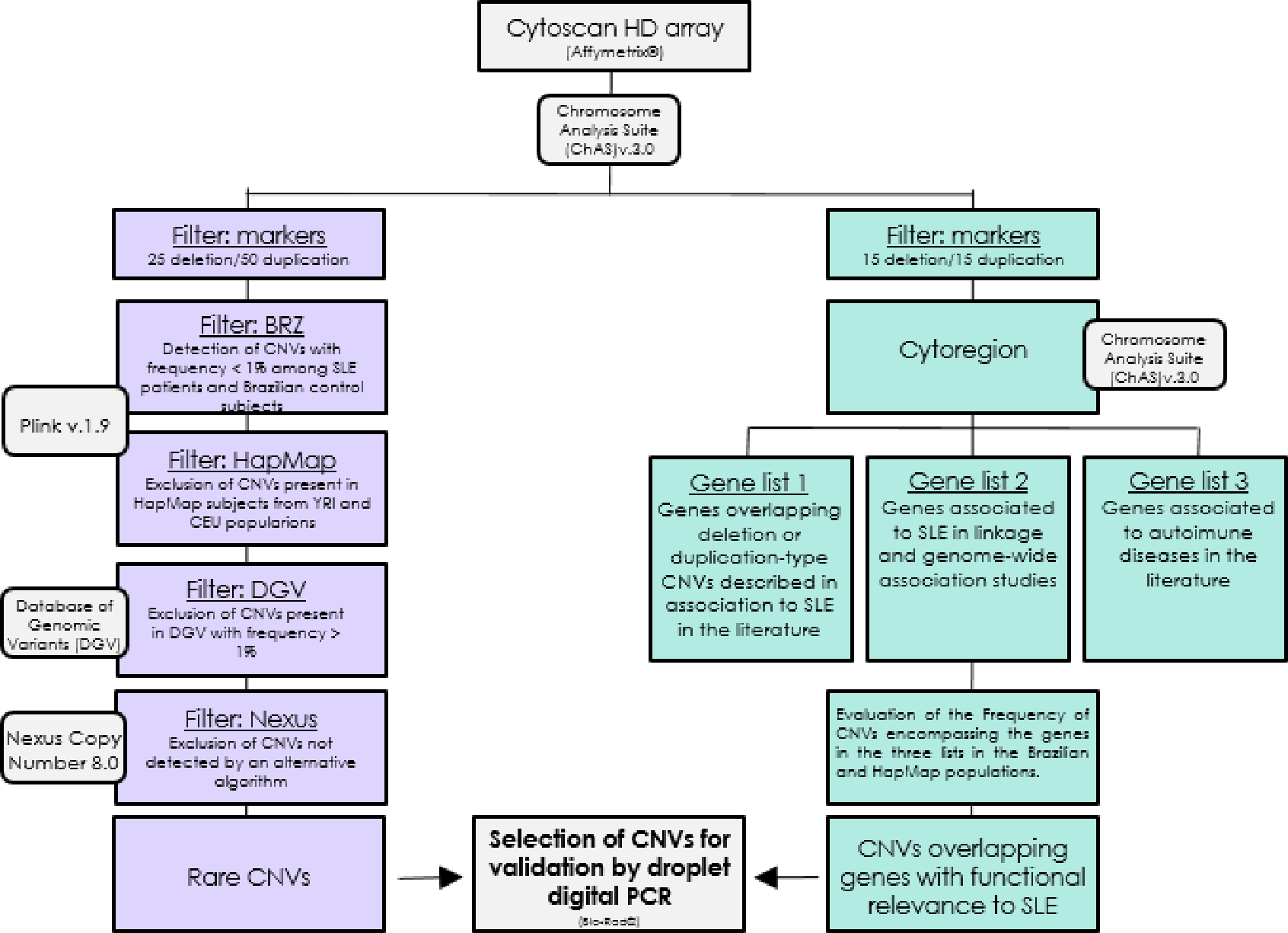

Supplement: S1 Fig — (TIF) [file pone.0206683.s001.tif]

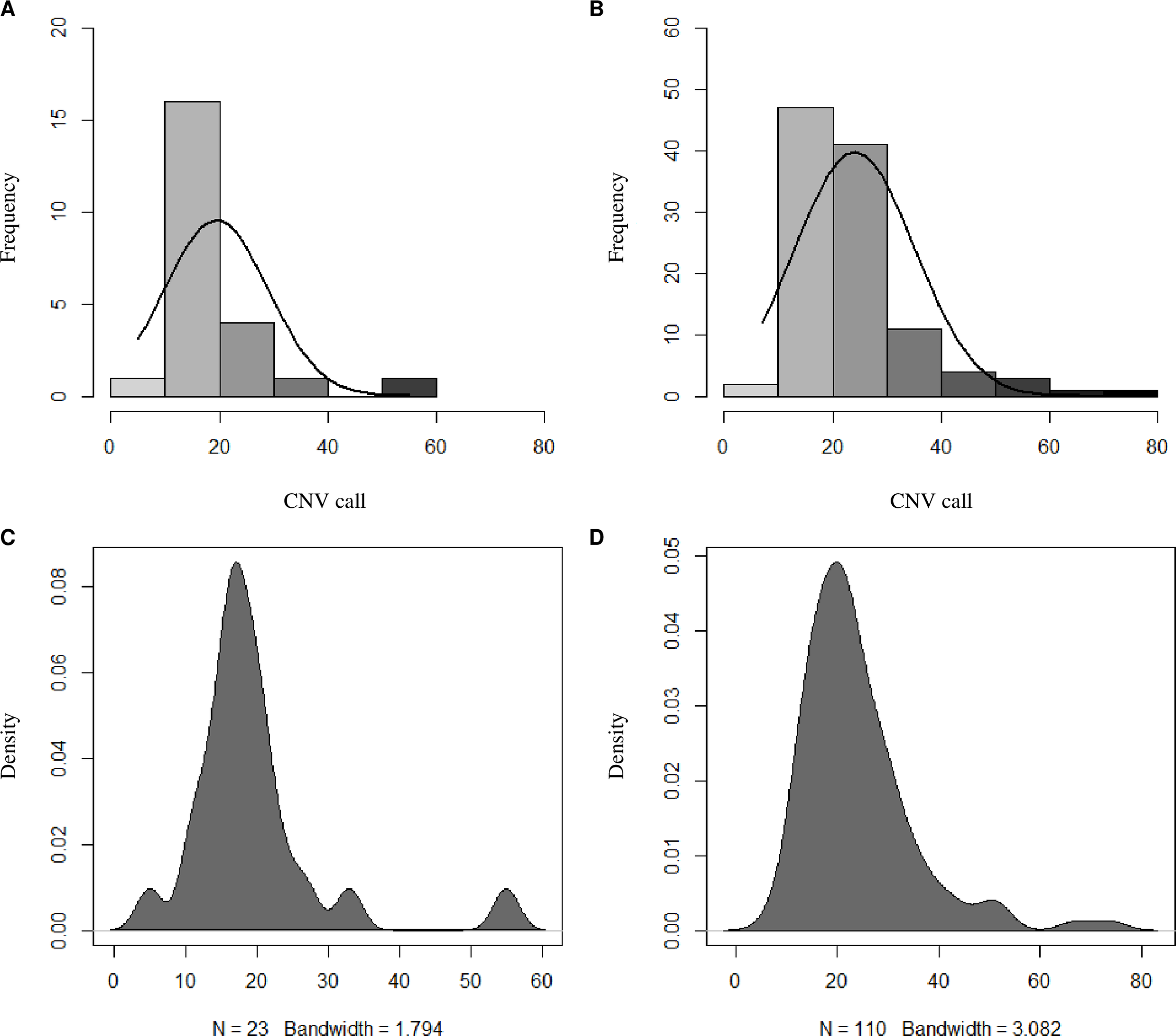

Supplement: S2 Fig — Histogram showing the frequency of each class of CNV call in systemic lupus erythematosus (SLE) patients (A) and in Brazilian controls (B). Kernel density plot showing the non-parametric distribution of probability density curve of CNVs in SLE patients (C) and in Brazilian controls (D). (TIF) [file pone.0206683.s002.tif]

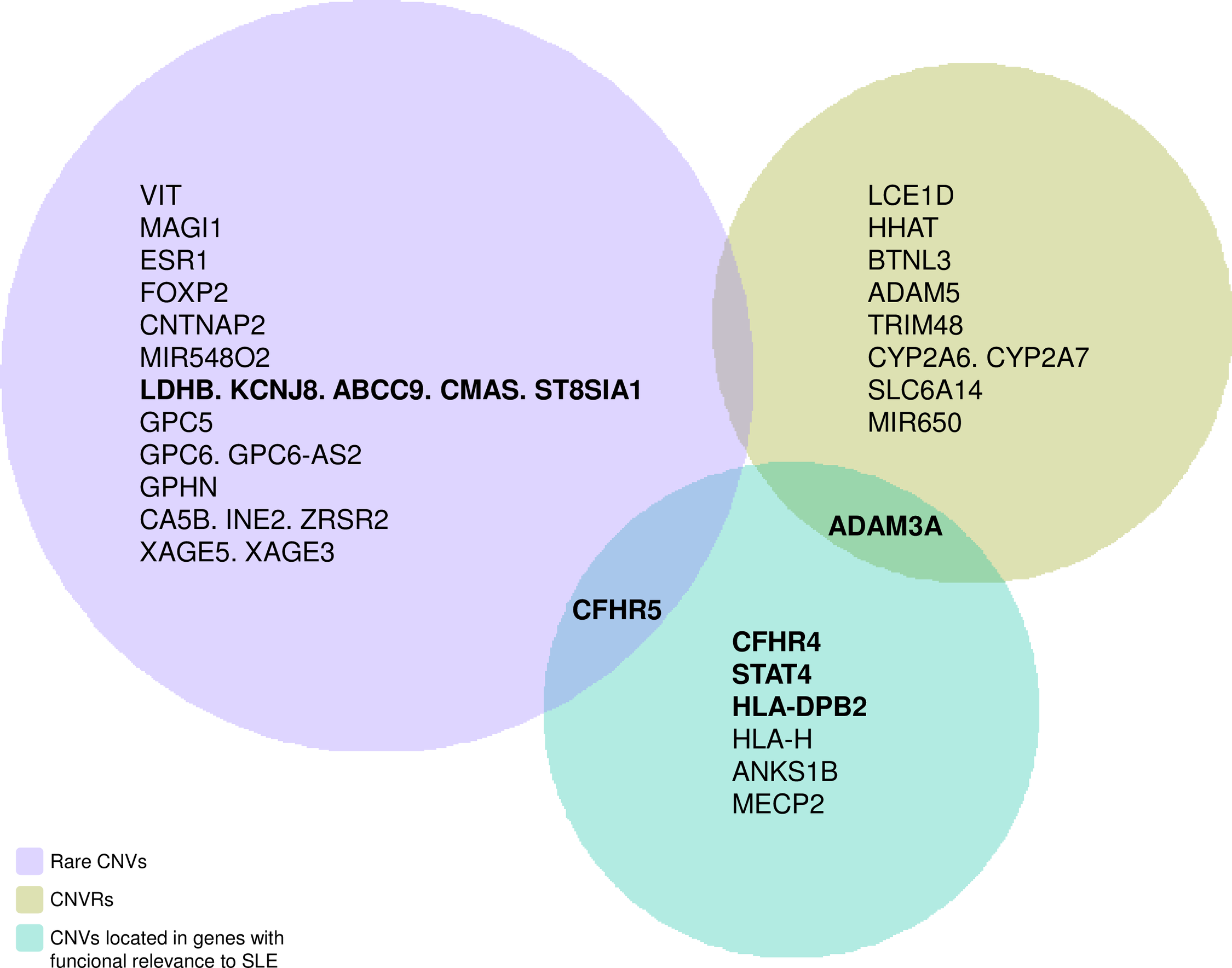

Supplement: S3 Fig — Genes in bold highlight those selected for further validation of the copy number status by target-specific methodology. (TIF) [file pone.0206683.s003.tif]

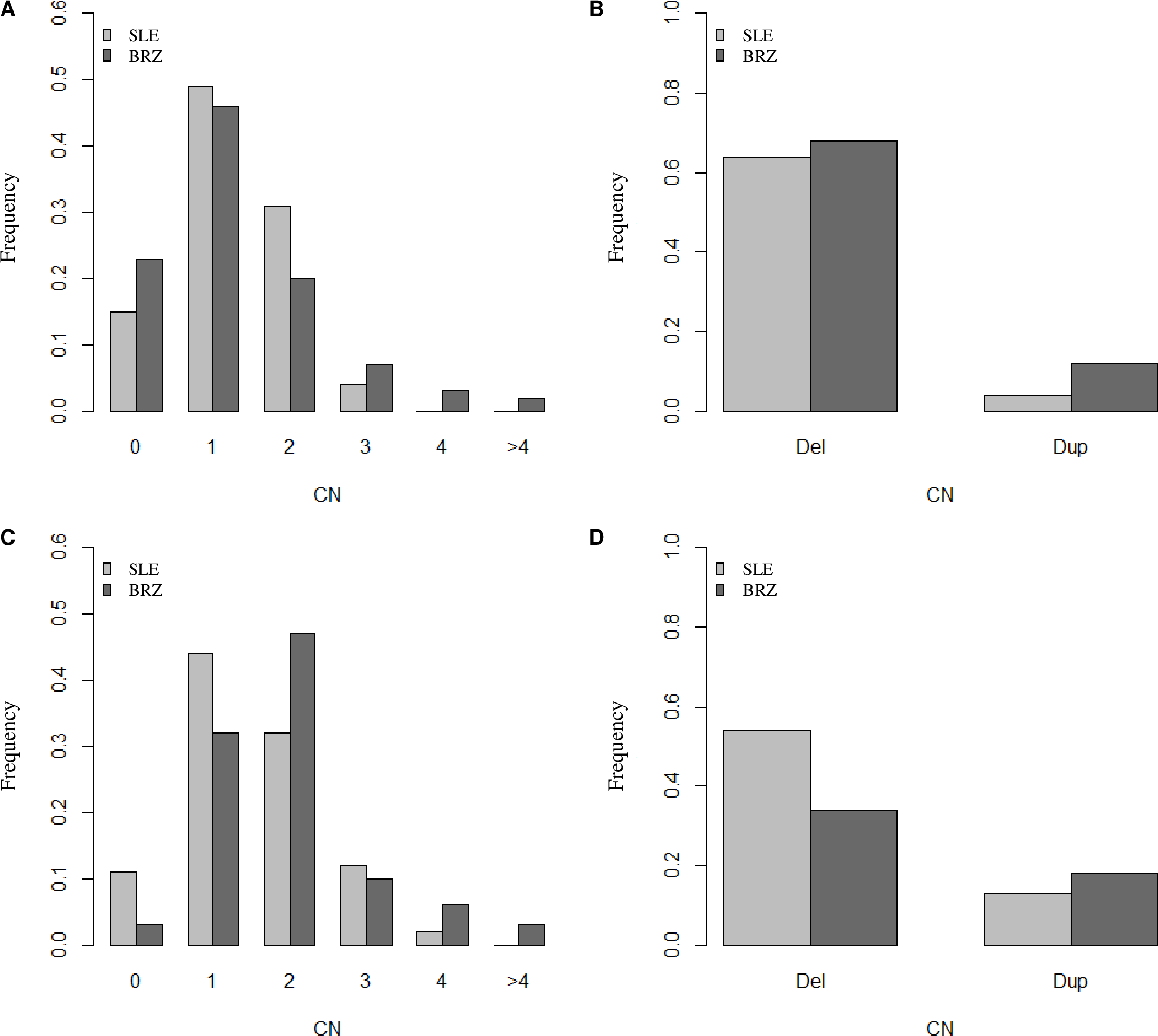

Supplement: S4 Fig — Frequency of copy number (CN), deletions (del) and duplications (dup) in ADAM3A (A, B) and FCGR3B (C, D) genes in systemic lupus erythematosus (SLE) and Brazilian control (BRZ) groups. (TIF) [file pone.0206683.s004.tif]

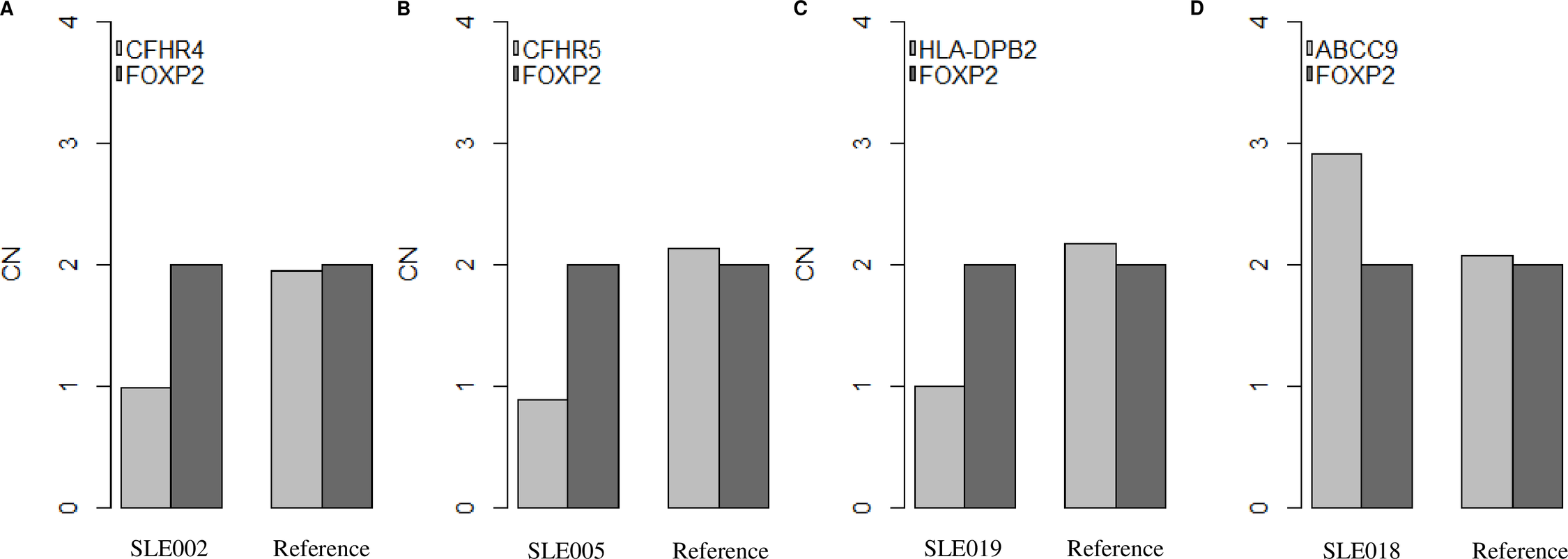

Supplement: S5 Fig — Charts showing the copy number (CN) for each SLE patient, confirming heterozygous deletions of CFHR4 in SLE002 patient (A), CFHR5 in SLE005 patient (B), HLA-DPB2 in SLE019 patient (C), and heterozygous duplication involving the LDHB, KCNJ8, ABCC9, CMAS and ST8SIA1 genes in the SLE018 patient (D). In all cases, the reference gene (FOXP2) shows invariable diploid status in the subjects analyzed. The reference sample confirmed the diploid copy number for both target and reference genes. (TIF) [file pone.0206683.s005.tif]
