# Supplementary material for: Copy number variation in the susceptibility to systemic lupus erythematosus
Source: PLoS One. 2018 Nov 28;13(11):e0206683. doi: 10.1371/journal.pone.0206683 (PMC6261406; doi:10.1371/journal.pone.0206683)
Supplement: S1 Table — (DOCX) [file pone.0206683.s006.docx]

**Table S1.** Primer sequences used in quantitative real-time PCR (qPCR) and droplet digital PCR (ddPCR).

| **Target** | **Forward primer 5’−3’** | **Reverse primer 5’−3’** |
| --- | --- | --- |
| *FOXP2** | TGACATGCCAGCTTATCTGTTT | GAGAAAAGCAATTTTCACAGTCC |
| *PAX6* | TTGCTTTCTGTGCGGTTGTG | AAGCTGCCAATGACTGAAGG |
| *FCGR3B** | CACCTTGAATCTCATCCCCAGGGTCTTG | CCATCTCTGTCACCTGCCAG |
| *ADAM3A* | AACACTTCAGGACAGCTTAGCC | GTTCCAGAGCTTTGTGAATGG |
| *CFHR4* | CGATCCAAGTCATCCCTAGAAG | AGGAGGAAACACCTGGAATC |
| *CFHR5* | CAAATTCATGTGCACTTTGTGG | CGCCCCATATCTTTGTATTTTG |
| *ABCC9* | GGAAAACTGGCAGGTGAAAC | TCTTCCTCATTCCCTTCACG |
| *HLA-DPB2* | TGAAGTTTGATTGCTTCTAGGC | TTACAATCCATTGGTGTAACAGG |
| *STAT4* | GCAGTCTGAGTACTCCGTTGAG | TGCCTTAGTTTCGAGGTCAG |

**primers described in Fanciulli et al. (2007) FCGR3B copy number variation is associated with susceptibility to systemic, but not organ-specific, autoimmunity. Nature genetics, 39, 721-723.*
