# Supplementary material for: Copy number variation in the susceptibility to systemic lupus erythematosus
Source: PLoS One. 2018 Nov 28;13(11):e0206683. doi: 10.1371/journal.pone.0206683 (PMC6261406; doi:10.1371/journal.pone.0206683)
Supplement: S2 Table — (DOCX) [file pone.0206683.s007.docx]

**Table S2.** Description of copy number variation identified in systemic lupus erythematosus (SLE) patients overlapping genes with functional relevance to SLE.

| **CNV** | | | | **Presence of CNVs (Del/Dup)** | | | | |
| --- | --- | --- | --- | --- | --- | --- | --- | --- |
| **Genomic location**  **(GRCh37/hg19)** | **Genes** | **Type** | **Size**  **(Kb)** | **SLE (n=23)** | **BRZ (n=110)** | **YRI (n=89)** | **CEU (n=85)** | **Pop**  **freq.** |
| chr1:196827841-196877037 | *CFHR4* | Del | 49 | 1/0 | 2/1 | 0/4 | 0/1 | > 1%/> 1% |
| chr1:196964969-196987806 | *CFHR5* | Del | 23 | 1/0 | 0/0 | 1/0 | 0/0 | < 1%/< 1% |
| chr2:191904170-191921930 | *STAT4* | Del | 18 | 1/0 | 0/0 | 0/0 | 0/0 | < 1%/< 1% |
| chr6:33083147-33095011 | *HLA-DPB2* | Del | 12 | 1/0 | 0/0 | 0/0 | 0/0 | < 1%/< 1% |
| chr6:29837187-29874629 | *HLA-H* | Del | 37 | 1/0 | 8/4 | 5/0 | 2/0 | > 1%/> 1% |
| chr12:99791778-99798721 | *ANKS1B* | Del | 7 | 6/0 | 25/0 | 9/0 | 8/1 | > 1%/< 1% |
| chr8:39247097-39386952 | *ADAM3A* | Del | 140 | 19/0 | 14/51 | 9/0 | 34/10 | ~50%/~20% |
| chrX:153334931-153335831 | *MECP2* | Dup | 1 | 0/7 | 0/28 | 0 | 0 | > 1%/> 5% |

*chr = chromosome; Del = deletion; Dup = duplication; ID = identification; Pop freq = population frequency; BRZ = Brazilian controls; YRI = Yoruba (HapMap project); CEU = Utah residents with Northern and Western European ancestry (HapMap project).*
