# Supplementary material for: Copy number variation in the susceptibility to systemic lupus erythematosus
Source: PLoS One. 2018 Nov 28;13(11):e0206683. doi: 10.1371/journal.pone.0206683 (PMC6261406; doi:10.1371/journal.pone.0206683)
Supplement: S3 Table — (DOCX) [file pone.0206683.s008.docx]

**Table S3.** Description of copy number variation regions (CNVRs) showing significant difference in frequencies between systemic lupus erythematosus (SLE) patients and Brazilian controls.

| **CNVR** | | | | **Frequency** | | | **Fisher’s Exact Test** | | **Pop freq** | |
| --- | --- | --- | --- | --- | --- | --- | --- | --- | --- | --- |
| **Genomic location**  **(GRCh37/hg19)** | **Genes** | **Type** | **Size (Kb)** | | **SLE** | **BRZ** | ***p*** | **OR [95% IC]** | **YRI** | **CEU** |
| chr1:72762662-72812440 | – | Del | 50 | | 0.00 | 0.16 | 4.17x10^-2^ | NA | 0.09 | 0.09 |
| chr1:152761909-152776589 | *LCE1D* | Del | 15 | | 0.09 | 0.30 | 3.82x10^-2^ | 0.22 [0.0-1.0] | 0.31 | 0.32 |
| chr1:210604732-210612839 | *HHAT* | Del | 8 | | 0.09 | 0.00 | 2.88x10^-2^ | NA | 0.00 | 0.00 |
| chr5:180378753-180442428 | *BTNL3* | Del | 64 | | 0.39 | 0.16 | 2.14x10^-2^ | 3.2 [1.1-9.6] | 0.23 | 0.24 |
| chr7:154720-158615 | – | Del | 4 | | 0.22 | 0.06 | 3.44x10^-2^ | 4.0 [0.9-16.7] | 0.03 | 0.04 |
| chr8:39213948-39389003 | *ADAM5, ADAM3A* | Del | 175 | | 0.83 | 0.59 | 3.52x10^-2^ | 3.3 [1.0-14.1] | 0.56 | 0.58 |
| chr11:48658169-48892181 | – | Del | 234 | | 0.09 | 0.00 | 2.88x10^-2^ | NA | 0.00 | 0.00 |
| chr11:54701631-55039246 | *TRIM48* | Del | 338 | | 0.13 | 0.01 | 1.64x10^-2^ | 15.8 [1.2-86.8] | 0.01 | 0.01 |
| chr14:22397988-22970492 | – | Del | 573 | | 0.13 | 0.45 | 4.65x10^-3^ | 0.2 [0.0-0.7] | 0.00 | 0.00 |
| chr19:41345129-41392187 | *CYP2A6, CYP2A7* | Del | 47 | | 0.22 | 0.05 | 1.41x10^-2^ | 5.7 [1.2-27.7] | 0.07 | 0.07 |
| chrX:115576235-115589080 | *SLC6A14* | Del | 13 | | 0.09 | 0.00 | 2.88x10^-2^ | NA | 0.00 | 0.00 |
| chr22:23151743-23183112 | *MIR650* | Dup | 31 | | 0.04 | 0.55 | 3.53x10^-6^ | 0.03 [0.0-0.2] | 0.14 | 0.14 |
| chrX:356232-387749 | – | Dup | 32 | | 0.17 | 0.02 | 8.30x10^-3^ | 11 [1.5-13.0] | 0.01 | 0.01 |
| chrX:395646-401509 | – | Dup | 6 | | 0.17 | 0.04 | 3.01x10^-2^ | 5.5 [0.9-32.1] | 0.00 | 0.00 |
| chrX:1793059-1818776 | – | Dup | 26 | | 0.13 | 0.01 | 1.64x10^-2^ | 15.8 [1.2-86.8] | 0.01 | 0.01 |

*chr = chromosome; OR = odds ratio; 95% CI = 95% confidence interval; Del = deletion; Dup = duplication; ID = identification; Pop freq = population frequency; BRZ = Brazilian controls; YRI = Yoruba (HapMap project); CEU = Utah residents with Northern and Western European ancestry (HapMap project).*
