# Supplementary material for: Copy number variation in the susceptibility to systemic lupus erythematosus
Source: PLoS One. 2018 Nov 28;13(11):e0206683. doi: 10.1371/journal.pone.0206683 (PMC6261406; doi:10.1371/journal.pone.0206683)
Supplement: S4 Table — (DOCX) [file pone.0206683.s009.docx]

**Table S4.** Description of copy number variation (CNVs) and copy number variation regions (CNVRs) selected for validation by target-specific methodology.

| **Genomic location**  **(GRCh37/hg19)** | **Genes** | **Type** | **CNV list** | **Methodology** |
| --- | --- | --- | --- | --- |
| chr8:39213948-39389003 | *ADAM5, ADAM3A* | Del | CNVR, functional CNV* | qPCR |
| chr1:161623196-161631963 | *FCGR3B* | Del | Literature | qPCR |
| chr1:196964969-196987806 | *CFHR5* | Del | Functional CNV, rare CNV | ddPCR |
| chr1:196827841-196877037 | *CFHR4* | Del | Functional CNV | ddPCR |
| chr2:191904170-191921930 | *STAT4* | Del | Functional CNV | ddPCR |
| chr6:33083147-33095011 | *HLA-DPB2* | Del | Functional CNV | ddPCR |
| chr12:21796951-22445614 | *LDHB, KCNJ8, ABCC9,*  *CMAS, ST8SIA1* | Dup | Rare CNV | ddPCR |

*chr = chromosome; Del= deletion; Dup = duplication; qPCR = quantitative real-time PCR; ddPCR = droplet digital PCR.*

**CNV located in genes with functional relevance to SLE.*
